# Maternal uniparental disomy for chromosome 6 in 2 prenatal cases with IUGR: case report and literature review

**DOI:** 10.1186/s13039-023-00670-0

**Published:** 2024-01-03

**Authors:** Yan Jiang, Yang Xue Xiao, Jiao Jiao Xiong, Victor Wei Zhang, Chang Dong, Lei Xu, Fang Liu

**Affiliations:** 1 Chongqing Health Center for Women and Children/Women and Children’s Hospital of Chongqing Medical University, Chongqing, China; 2 AmCare Genomics Lab, Guangzhou, China

**Keywords:** Uniparental disomy, Intrauterine growth restriction, Prenatal diagnosis, Maternal UPD6

## Abstract

**Background:**

Uniparental disomy (UPD) is a rare genetic condition leading to potential disease risks. Maternal UPD of chromosome 6 upd(6)mat is exceptionally rare, with limited cases reported. This study reported two new cases of upd(6)mat and reviewed the literature of previous cases.

**Case presentation:**

Both cases exhibited intrauterine growth restriction (IUGR), and genetic analysis confirmed upd(6)mat in each case. The literature review identified a total of 19 cases. IUGR and preterm labor were the most common two symptoms observed, and additional anomalies and genetic variations were also reported in some cases.

**Conclusion:**

upd(6)mat is potentially associatied with IUGR, but the precise genotype–phenotype relationship remains unclear. The cases with upd(6)mat may present clinical features due to imprinting disorders.

## Introduction

Uniparental disomy (UPD) is a situation where both homologous chromosomes are inherited from a single parent [1]. The mechanisms leading to UPD include trisomy rescue, monosomy rescue, mitotic crossing over, and chromosomal rearrangements [2]. UPD manifests in two forms: isodisomy, characterized by two identical copies of the chromosome inherited from one parent, and heterodisomy, where both homologous chromosomes originate from one parent. UPD can cause diseases by increasing the risk of autosomal recessive diseases or imprinted disorders [3]. Imprinted regions, regulated by epigenetic mechanisms such as DNA methylation, encompass genes that express in parent-of-origin specific patterns. The occurrence of UPD can result in the overexpression or silencing of imprinted genes.

Specific phenotypes of UPD have been established in maternal UPD for chromosomes 7, 11, 14, 15, and 20 [2] and paternal UPD for chromosomes 6, 11, 14, 15, and 20. On the other hand, it is still disputable for some chromosomes whether UPD has phenotypic effects attributable to imprinted disorders. Segmental UPD refers to a situation where only a part of a chromosome is inherited from one parent, while the rest is biparental. Segmental UPD can be either heterodisomic or isodisomic. Mixed hetero- and isodisomy combines heterodisomy and isodisomy within the same chromosome [4]. Maternal UPD of chromosome 6 is scarce, with less than 20 cases reported to date. The clinical presentation of maternal UPD6 is variable. Intrauterine growth restriction (IUGR) and preterm labor were the most common presentation observed. No clear phenotype-genotype correlation has been established until now. It is also worth noting that the presence of (hidden) mosaicism for trisomy 6 poses a potential risk, and its impact on the phenotype should be considered in the analysis of UPD6 cases [5].

This study presents two new prenatal cases of maternal UPD6, both of which exhibited IUGR. The aim of this study was to contribute new cases to the existing literature on this rare genetic condition. Furthermore, we conducted a comprehensive review of previous literature to summarize the cases of maternal UPD6.

## Case presentation

### Case 1

**Fig. 1:**
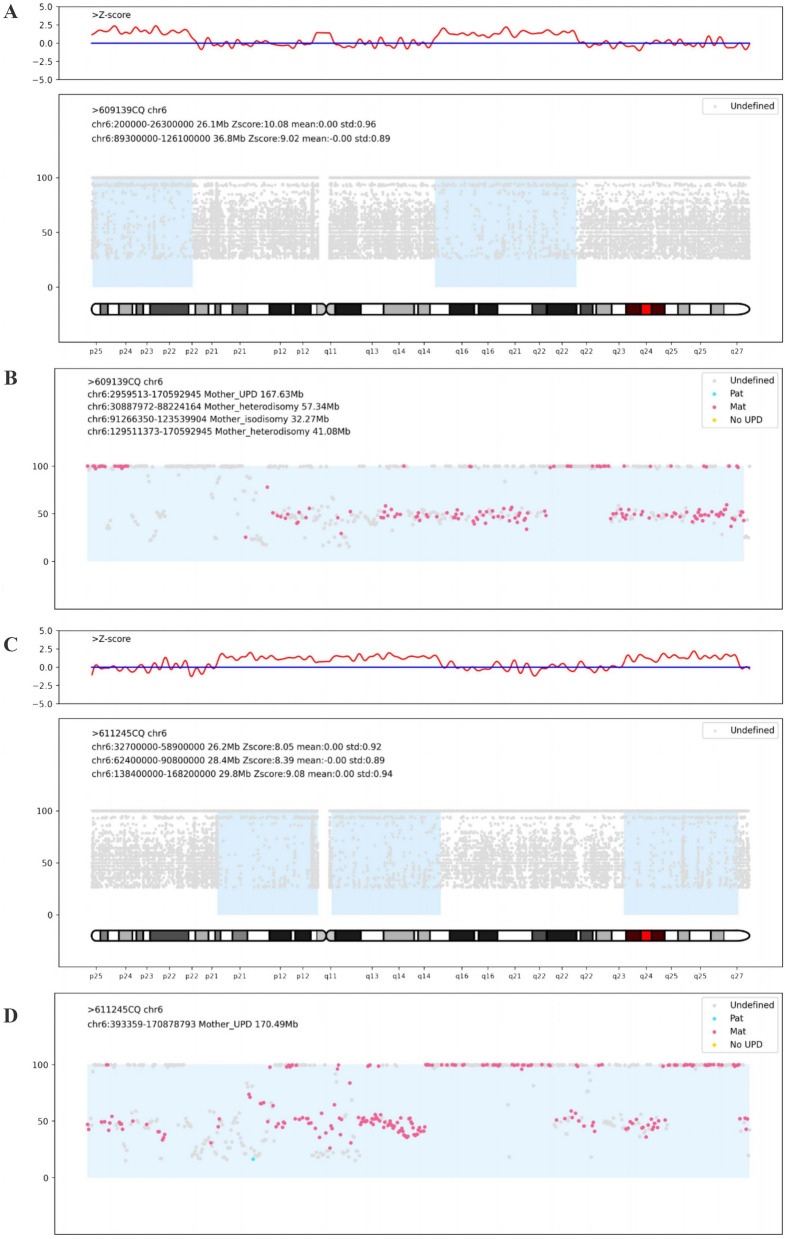
UPD for chromosome 6 in two patients. Medium coverage whole genome sequencing showed AOH regions of chromosome 6 for Case 1 (**A**) and Case 2 (**C**). Clinical exome sequencing showed the UPD chromosome 6 had both isodisomy and heterodisomy for Case 1 (**B**) and Case 2 (**D**) The AOH scatter plots and the Z-score distribution of chromosome 6 were shown. Z-scores were calculated for each 25 kb window for each sample across the batch. Marked AOH regions when at least four consecutive windows had Z-scores below − 2 or above 2

The mother was 33 years old, gravida 3 para 1, and the father was 34 years old. The couple was non-consanguineous and belonged to the Chinese Han population. The mother had a Hepatitis B Virus (HBV) infection. She gave birth to a healthy girl and had one abortion due to non-medical reasons. Fetal growth, as assessed during nuchal translucency (NT) screening at 12 + 6 gestational weeks (gw), was consistent with the expected measurements (Crown-Rump Length (CRL): 66.8 mm), and the NT was within a normal range (1.5 mm). The non-invasive prenatal testing (NIPT) plus test at 17 gw showed a low risk of chromosome aneuploidy. At 23 gw, ultrasound screening revealed signs of intrauterine growth retardation (IUGR), indicated by Biparietal Diameter (BPD): 5.37 cm (− 0.88SD), Head Circumference (HC): 20.18 cm (− 1.606SD), Abdominal Circumference (AC): 17.15 cm (− 1.606SD), Femur Length (FL): 3.77 cm (− 0.868SD), and an estimated fetal weight of 477 g (6.6%). At 30 gw, amniocentesis was performed for genetic testing and fetal magnetic resonance imaging (MRI) was performed for fetal structural examination. The FISH (Fluorescence in situ hybridizatio)analysis of the fetus did not indicate 13, 18, 21 and sex chromosome aneuploidy abnormalities. We utilized chromosome analysis by medium coverage whole genome sequencing (CMA-seq), a method optimized for reliable the absence of heterozygosity (AOH) detection, revealing the AOH of 26.1 Mb at 6p25.3p22.2 (200000-26300000) and 36.8 Mb at 6q15q22.31 (89300000-126100000) on chromosome 6, based on the hg19 human genome reference. Clinical exome-sequencing (CES) identified maternal UPD of at least 167.63 Mb (2959513-170592945) on chromosome 6. The heterodisomy UPD regions included 57.34 Mb at chr6: 30887972-88224164 and 41.08 Mb at chr6: 129511373-170592945. The isodisomy UPD region included 37.27 Mb at chr6: 91266350-123539904 (Fig. 1), all based on the hg19 reference. No pathogenic copy number variants (CNVs) or single nucleotide variants (SNVs) were detected in the fetus. Fetal MRI confirmed the ultrasound findings and did not reveal any additional malformations. IUGR was persistent at 33 gw, and ultrasound showed BPD 7.85 cm (− 2.45SD), HC 28.02 cm (− 2.67SD), AC 25.02 cm (− 3.14SD), FL 5.66 cm (− 2.08SD), and estimated fetal weight 1425 g (0.2%). Consequently, the parents elected to terminate the pregnancy due to severe IUGR. Post-mortem and examination of the placenta were declined.

### Case 2

The mother was 32 years old, gravida 2 para 1, and the father was 33 years old. The couple was non-consanguineous, and both were Chinese Han populations. The mother presented with a decreased mean corpuscular volume (MCV), and thalassemia gene screening yielded the expected results. Her previous child was a healthy boy. Fetal ultrasound at 29 gw revealed the following measurements: BPD: 6.63 cm (− 2.668SD), HC: 24.81 cm (− 3.139SD), AC: 21.95 cm (− 2.842SD), FL: 5.13 cm (− 1.31SD), indicating IUGR. The ultrasound also detected a cystic mass in the lower right abdomen, raising suspicion of intestinal duplication. Amniocentesis and fetal MRI were performed on the same day. The FISH analysis of the fetus did not indicate 13, 18, 21 and sex chromosome aneuploidy abnormalities. CMA-seq identified three regions of AOH: 26.2 Mb at 6p21.32p11.1 (chr6:32700000-58900000), 28.4 Mb at 6q11.1q15 (62400000-90800000), and 29.8 Mb at 6q23.3q27 (138400000-168200000). Clinical Exome Sequencing (CES) revealed maternal UPD of at least 170.49 Mb (393359-170878793) at chromosome 6. The heterodisomy regions included 31.98 Mb at chr6:393359-32370927 and 91.27 Mb at chr6:91266350-138196066. The isodisomy UPD region included 16.57 Mb at chr6:33408632-88224164 and 14.96 Mb at chr6:152469188-167424293 (Fig. 1). No pathogenic copy number variants (CNVs) or single nucleotide variants (SNVs) were detected in the fetus. Fetal abdominal MRI findings were consistent with the ultrasound results. The pregnancy was terminated, and a post-mortem examination was declined.All participants provided informed consent prior to their inclusion in the study.

## Literature review

The literature search was conducted using PubMed (https://pubmed.ncbi.nlm.nih.gov) with the search terms "Maternal uniparental disomy chromosome 6" or "Maternal UPD6". A total of 19 cases, including 2 cases from our study, were summarized from 15 articles [6–19]. The following information of each case was reviewed: sex, prenatal/postnatal phenotypes, placenta findings, size of UPD, type of UPD, karyotype, and other genetic findings (Table 1).

**Table 1:** Overview on the UPD(6)mat patients reported in the literature

References	ID	Sex	Size of UPD6	Type	Other genetic findings	Prenatal	Postnatal	Placenta
van den Berg-Loonen et al. [6]	1	M	Whole chromosome 6	Isodisomy	Not detected	IUGR	1. Low birth weight	NR
							2. Developmental delay	
							3. Sarcoidosis	
							4. Renal insufficiency	
Spiro et al. [7]	2	F	Whole chromosome 6	Isodisomy	*CYP21A2*: (p.I172N)	IUGR	CAH	Normal
Cockwell et al. [9]	3	M	Whole chromosome 6	Heretodisomy	mos46,XY[13]/47,XY, + 6[12]	1. Exomphalos	Intrauterine death	Normal
						2. AVSD		
Parker et al. [8]	4	M	Unknown	Isodisomy	1. 48,XXY, mar[30]/47,XXY[20]	1. IUGR	1. Klinefelter Syndrome	NR
					2. *CYP21A2* whole gene deletion, hom	2. Oligohydramnios	2. CAH	
					3. UPD X(mat)		3. CHD	
							4. Developmental delay	
Haag et al. [10]	5	F	Unknown	Unknown	mos46,XX[?]/47,XX, + 6[?]	1. IUGR	NR	NR
						2. CHD		
Gumus et al. [11]	6	M	1. 6p21.1-6p24.3	Isodisomy	*MOCS1*: 217C > T (p.R73W), hom	Dandy–Walker malformation	Molybdenum cofactor deficiency	NR
			2. 6q13-6q22.31					
Salahshourifar et al. (2010)	7	M	Whole chromosome 6	Heterodisomy	Not detected	Unilateral cleft lip and palate	Unilateral cleft lip and palate	NR
Sasaki et al. [12]	8	M	Whole chromosome 6	Isodisomy& heterodisomy	*CUL7*: c.2975G > C (p.R992P), hom	IUGR	1.Severe developmental delay	NR
							2. 3M-syndrome	
Roosing et al. [13]	9	M	Whole chromosome 6	Isodisomy	*TULP1*: c.1258C > A (p.Arg420Ser),hom	NR	1. Low birth weight	NR
							2. Cone dystrophy	
Poke et al. [14]	10	F	6q16.1qter	Isodisomy	Not detected	1.IUGR	1. Premature(35w), low birth weight (< 10%)	1. Placental insufficiency
						2. Reduced fetal movements	2. Dysphagia	2. Abnormal villous maturation
							3. Gastro-esophageal reflux	
							4. Developmental delayed (before 2y)	
Takimoto et al. [15]	11	F	Whole chromosome 6	Isodisomy	*WASP*:c.1276_1285 del_GCCCCTGGTG(p.A426Gfs*15), het	NR	1.Premature(29w), low birth weight (< 10%)	NR
							2.Wiskott-Aldrich Syndrome	
Lazier et al. [16]	12	M	Whole chromosome 6	Isodisomy	Not detected	1. IUGR (19w)	1. Low birth weight	Placental dysfunction
						2. Ambiguous genitalia	2. Abnormal genitalia	
							3.Respiratory distress syndrome	
Leung et al. [17]	13	F	Whole chromosome 6	Isodisomy& heterodisomy	Not detected	1. IUGR	Premature (34w)	mos47,XX + 6[12]/46,XX[19]
						2. Oligohydramnios		
	14	F	Whole chromosome 6	Heterodisomy	Not detected	IUGR	Premature (32w)	mos47,XX + 6[14]/46,XX[16]
Eggermann et al. [18]	15	F	6p12.3-6q27	Isodisomy& heterodisomy	Not detected	IUGR	1. Facial dysmorphism	Placental dysfunction
							2. Developmental delay	
							3. Language delay	
	16	M	6p25.3-6q27	Isodisomy& heterodisomy	*CYP21A2*: exon 1–8 deletion, hom	1. IUGR	1. CAH	NR
						2. Oligohydramnios	2. Developmental delay	
Kerr et al. [19]	17	M	Whole chromosome 6	Isodisomy	Not detected	NR	1. Premature (32w)	NR
							2. Developmental delay (before 14m)	
Our case	18	M	Whole chromosome 6	Isodisomy& heterodisomy	Not detected	IUGR	TOP	NR
Our case	19	F	Whole chromosome 6	Isodisomy& heterodisomy	Not detected	1. IUGR	TOP	NR
						2. Abdominal cyst		

*CAH* congenital adrenal hyperplasia, *CHD* congenital heart disease, *F* female, *M* male, *NR* not reported, *IUGR* intrauterine growth retardation, *hom* homozygous, *TOP* termination of pregnancy

Prenatal conditions were mentioned in 16 cases, while the other 3 cases lacked description. Among the cases with available information, 13 cases (68.4%) presented with IUGR, the most common prenatal symptom. Ten cases (19.0%) combined with other anomalies, including oligohydramnios and malformations in the brain, heart, face, or genitalia. Preterm and low birth weight was reported in 8 cases (42.1%), and developmental delay were shown in 7 cases (36.8%). Postnatal phenotypes varied considerably due to the range of ages at the time of reporting, spanning from neonatal to 52 years old, as well as the specific diagnoses of associated diseases. Placental findings were not documented in the majority of cases. However, placental insufficiency was reported in 3 cases (15%), and mosaic trisomy 6 was observed in 2 cases (10.5%).

Ten cases (52.6%) were identified as isolated upd(6)mat without other genetic findings. Six cases (36.8%) had autosomal recessive (AR) diseases due to isodisomy maternal UPD6 involving CYP21A2, MOCS1, CUL7, and TULP1. One case (5.3%) had an X-linked disease due to a heterozygous variant in Wiskott-Aldrich Syndrome Protein (WASP). Three cases (15.8%), including one case with AR disease, exhibited mosaic chromosomal aneuploidies involving chromosome 6 or chromosome X. The size of maternal UPD6 mainly affected whole chromosome 6 (42.1%).

## Discussion

UPD has an overall prevalence of 1 in 2000 births, in which the prevalence of disease-associated UPD is approximately 1 in 3500–5000 [20, 21]. upd(6)pat is associated with neonatal transient diabetes. Historically, upd(6)mat was considered a rare occurrence, with only 19 reported cases worldwide since 1996. Among the total 19 cases, 13 reported IUGR, 3 cases did not present IUGR, and 3 cases whose prenatal condition were not mentioned. One case (Case 9) reported low birth weight, which indicates IUGR, while the other 2 cases (Case 11 and 17) were both preterm. Thus, the proportion of IUGR in upd(6)mat cases might be higher than previously assumed. Recent research by Mackay et al. [22] has emphasized the previously underestimated significance of upd(6)mat in imprinting diagnostics, especially concerning Silver-Russell syndrome (SRS). The widely used commercial diagnostic kit for SRS has been instrumental in the global diagnosis of children with suspected SRS. This has led to the discovery of numerous upd(6)mat cases in major diagnostic labs, often attributing growth restriction to this UPD. Their study highlighted seven such cases detected across nine diagnostic labs, suggesting that upd(6)mat is emerging as a more frequent related diagnosis, even surpassing upd(20)mat, challenging previous notions of its rarity.

In China, IUGR is one of the prevalent reasons for delayed termination of pregnancy. upd(6)mat, as a rare genetic anomaly, presents unique challenges in the Chinese context. The intersection of medical complexities and cultural factors may significantly influence parental attitudes and decisions toward fetal anomalies, highlighting the need for a comprehensive approach that considers both medical and cultural impacts on family decision-making.

When isodisomy occurs, the entire chromosome shows an absence of heterozygosity, significantly increasing the risk of autosomal recessive (AR) diseases. According to the exome-based sequencing results, there was no overlap of isodisomy regions in our two cases. Furthermore, clinical exome sequencing for genes with definite corresponding diseases collected from the Online Mendelian Inheritance in Man (OMIM) database revealed no suspicious variants that could account for IUGR. These results substantially diminished the possibility that AR diseases accounted for IUGR in these two cases. In our study, we provided NIPT results for only one case, indicating a low possibility of mosaic trisomy 6 in the placenta. However, we acknowledge that a normal NIPT result cannot definitively exclude the presence of low-level mosaicism in the placenta. Furthermore, while karyotyping of placenta can offer insights into the presence of mosaic trisomy 6, we did not conduct this analysis in our study. This omission limits our understanding of the potential contribution of an underlying mosaic trisomy 6 cell line in the placenta to the observed growth restriction.

Considering the genetic test results and NIPT results, we highly suspected that upd(6)mat was responsible for IUGR in these two cases. It is important to note that while in many known imprinting disorders, paternal UPD is often associated with overgrowth and maternal UPD with growth retardation, there are exceptions. Specifically, paternal UPD 6 has been observed to result in IUGR/postnatal growth retardation(PNGR). For instance, Temple syndrome associated with UPD(14)mat [23], Russell-Silver syndrome associated with UPD(7)mat or UPD(11)mat [24, 25], and Mulchandani-Bhoj-Conlin syndrome caused by UPD(20)mat [24–26], all result in prenatal and postnatal growth retardation. Conversely, UPD(14)pat, associated with Kagami-Ogata syndrome, is a severe overgrowth syndrome characterized by fetal macrosomia, polyhydramnios, and abdominal wall defects. Paternal UPD11 is also associated with fetal overgrowth, identified as Beckwith-Wiedemann syndrome (BWS) [2]. Among the upd(6)mat cases in the literature, most of the UPDs encompass the entire chromosome 6 or involve the known imprinting region 6q24.2. Cases 15 and 16 received methylation-specific single-nucleotide primer extension (MS-SNuPE) and methylation-specific multiplex ligation-dependent probe amplification (MLPA) for PLAGL1 and IGF2R differentially methylated regions (DMRs) in 6q24. The results from these cases showed hypermethylation in these regions compared with normal controls, indicating an imprinting defect. In the broader context of chr6, beyond PLAGL1, there are other imprinted genes. Some of these, as detailed by Court et al. [27], are germline-imprinted, while others, as described by Hanna et al. [28], are placental. While the phenotype of upd(6)mat remains somewhat unclear, the association of upd(6)mat with certain imprinting defects, such as the hypermethylation of PLAGL1 TSS-DMR, is well-established. As sequencing technology advances in its application for disease diagnosis and prenatal diagnosis, more cases of upd(6)mat will likely be identified, further solidifying the genotype–phenotype correlation. For future upd(6)mat cases, systemic methylation tests such as methylation sequencing are recommended to uncover the full spectrum of imprinting defects caused by upd(6)mat.

Contrary to previous assumptions, upd(6)mat is not as rare as once believed. It has gained recognition in imprinting diagnostics and is even considered a potentially related disorder to SRS. The widely-used commercial diagnostic kit for Silver-Russell syndrome (SRS) facilitates the global diagnosis of children with suspected SRS. Many diagnostic labs have identified cases of upd(6)mat, often linking this upd to growth restriction. A recent study by Mackay et al. highlighted seven such cases detected across nine diagnostic labs, suggesting that upd(6)mat is a more frequent related diagnosis than previously thought, even surpassing upd(20)mat.

In conclusion, upd(6)mat mainly manifests as IUGR and preterm labor. It is hypothesized that upd(6)mat may contribute to IUGR through imprinting defects. Nevertheless, based on the available information from existing cases, the precise association between upd(6)mat and IUGR, as well as other symptoms, cannot be definitively determined, necessitating further research with a larger sample size. It is also crucial to consider the limitations of NIPT results, especially when evaluating the potential of low-level mosaicism in the placenta.
